# Expression of Transient Receptor Potential Channel Genes and Their Isoforms in Alpha-Cells and Beta-Cells of Human Islets of Langerhans

**DOI:** 10.1155/2022/3975147

**Published:** 2022-08-03

**Authors:** Gabriel M. Matos, Björn Andersson, Md. Shahidul Islam

**Affiliations:** ^1^Department of Cell and Molecular Biology, Karolinska Institutet, Stockholm, Sweden; ^2^Department of Clinical Science and Education, Södersjukhuset, Karolinska Institutet, Stockholm, Sweden; ^3^Department of Emergency Care and Internal Medicine, Uppsala University Hospital, Uppsala, Sweden

## Abstract

Expression of the transient receptor potential (TRP) channel genes and their isoforms in the alpha-cells and the beta-cells of the human islets of Langerhans has not been studied in detail. In this study, we have analyzed the RNA sequencing data obtained from purified human alpha-cells and beta-cells to identify the genes and their isoforms that are expressed differentially in these two cell types. We found that *TRPC1*, *TRPC4*, *TRPC7*, *TRPM3*, and *TRPML1* were differentially expressed in these two cell types. *TRPC1*, *TRPM3*, and *TRPML1* were expressed at a higher level in the beta-cells than in the alpha-cells. *TRPC4* and *TRPC7* were expressed at a higher level in the alpha-cells than in the beta-cells. The *TRPC4-206* isoform was expressed at a 45-fold higher level in the alpha-cells compared to the beta-cells. Expression of *TRPM3-202* was 200-fold and *TRPM3-209* was 25-fold higher in the beta-cells than in the alpha-cells. Our study has demonstrated the relative abundance of expression of the TRP channel genes and their isoforms in the human alpha-cells and the beta-cells.

## 1. Introduction

The pancreatic islets of Langerhans contain three major types of cells, the *α*-, *β*-, and *δ*-cells that secrete glucagon, insulin, and somatostatin, respectively [1]. Other minor cell types of islets are the ghrelin-producing *ϵ*-cells and the pancreatic polypeptide cells [2, 3]. The endocrine cells of islets secrete more than just the main peptide hormone products [2, 3]. In the human islets, the relative abundance of the *α*-, *β*-, and *δ*-cells is 10-65%, 28-75%, and 1.2-22%, respectively [4]. However, the estimated percentages of different cell types differ considerably between studies [5–7]. Impairment in the normal process of secretion of insulin and glucagon from the islet cells is involved in the pathogenesis of diabetes, a major public health problem throughout the world [8]. The molecular mechanisms involved in the secretion of insulin from the *β*-cells, and glucagon from the *α*-cells include intermediary metabolism of nutrients and participation of many ion channels [8]. In this respect, the roles of several ion channels of the transient receptor potential (TRP) family have received considerable attention [9–11].

We have reported which TRP channel genes are expressed in the human *β*-cells [12]. In that study, we analyzed RNA-seq (RNA sequencing) data obtained from *β*-cells of only two human donors. The purity of *β*-cells used in that study was not high. We did not include the TRP channel genes that were expressed at low levels because we used an arbitrary cut-off point as an expression threshold by which we described whether a gene was expressed or not [12]. We examined the expression of the TRP channel genes only in the *β*-cells, but not in the *α*-cells.

In this study, we have analyzed the RNA-seq data from highly purified human *α*-cells and *β*-cells obtained from seven human donors. The purification of human *α*-cells and *β*-cells and the initial RNA-seq analysis were done by Blodgett et al. [13]. We have identified the TRP channel genes, including those that are expressed at low level, in these two main types of cells of human islets of Langerhans. We have also identified the different splice variants of these genes that are expressed in these cells. In addition, we have identified the TRP channel genes and their isoforms that are differentially expressed in these two types of cells.

## 2. Materials and Methods

For identifying the TRP channels and their splice variants, we analyzed publicly available RNA-seq data obtained from highly purified human pancreatic *α*-cells and *β*-cells. The methods for purification of the human *α*-cells and *β*-cells and the protocols for RNA-sequencing have been described in detail by Blodgett et al. [13]. In short, islets isolated from adult, healthy, deceased humans were dissociated into single cells, fixed, permeabilized, and stained by anti-insulin, anti-glucagon, and anti-somatostatin antibodies, and highly purified (>97% pure) *α*-cells and *β*-cells were sorted by fluorescence activated cell sorter using gating strategies described before [13]. RNA was extracted from 6 *α*-cell preparations and 7 *β*-cell preparations from 7 adult donors (5 males, 1 female, and 1 undefined) of variable ages (4–60 years), and body mass index (BMI) (21.5–37 kg/m^2^). Libraries were constructed by RNA fragmentation, first- and second-strand DNA synthesis, ligation of adaptors, amplification, library validation, and removal of ribosomal RNA. Illumina HiSeq 2000 platform was used for 91 base pair paired-end sequencing and 20 million clean reads per sample were acquired [13].

We downloaded the RNA-seq data from the European Nucleotide Archive (https://www.ebi.ac.uk/ena/data/view/PRJNA280220). We assessed the reads quality by using FastQC (https://www.bioinformatics.babraham.ac.uk/). We mapped the data against the human mitochondrial genome (https://www.ncbi.nlm.nih.gov/nuccore/NC_012920.1) by using Bowtie 2 and removed from analysis any mapped reads corresponding to the mitochondrial reads (option-un-conc-gz) [14]. The software STAR (Spliced Transcripts Alignment to a Reference, v2.7.3) was used for mapping (standard RSEM parameters for STAR) [15]. We mapped the filtered reads to the annotated human genome (version GRCh38.p13). We normalized the resulting expression counts by RSEM (RNA-seq by Expectation-Maximization) to TPM values (transcripts per million).

For analyzing differential expression of the genes and the isoforms, we used EBSeq R-package with a false discovery rate (FDR) < 0.01. Only genes and isoforms with PPDE (posterior probability of differential expression) greater than 0.99 were considered differentially expressed [16].

We used mean TPM cut-off of 0.5 to consider whether a gene is expressed or not in each cell population; mean TPM values < 0.5 were transcriptional noise. By low level expression, we meant TPM values < 3.

## 3. Results

### 3.1. Expression of TRP Channel Genes in the *β*-Cells

**Figure 1:**
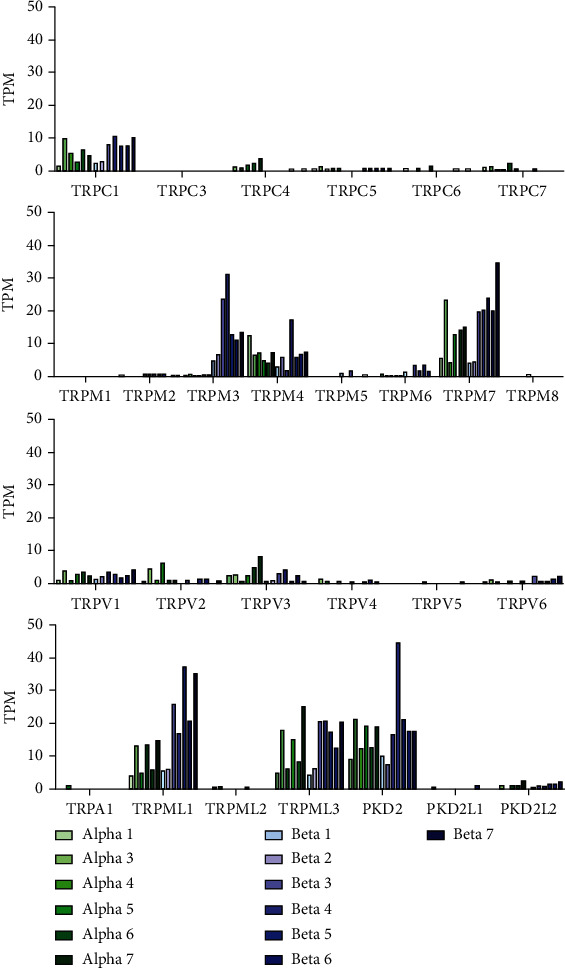
Expression of the TRP channels belonging to different families. The expression profiles are shown as bar plots on transcripts per kilobase million (TPM) scale. The figure shows the relative levels of expressions of the channels of the TRPC family (a), TRPM family, and TRPV family. (b) TRPA1, members of the TRPP and TRPML. TRPML1 = MCOLN1, TRPML2 = MCOLN2, TRPML3 = MCOLN3, PKD2 = TRPP1, PKD2L1 = TRPP2, PKD2L2 = TRPP3. Data obtained from the *α*-cells are shown in different shades of green bars, and those from the *β*-cells are shown in different shades of blue bars.

Among the TRPC (transient receptor potential canonical) channel genes, TRPC1 was expressed at high level in all samples of *β*-cells (Figure 1(a)). TRPC5 was expressed at low levels in 5 out of 7 samples of *β*-cells. TRPC3 was not expressed in the *β*-cells. We observed expression of TRPC4 at low level in only 1 sample of *β*-cells. TRPC6 and TRPC7 were expressed at low levels in only 2 and 1 sample of *β*-cells, respectively.

Among the TRPM (transient receptor potential melastatin) channel genes the expression of TRPM3, TRPM4, and TRPM7 in the *β*-cells was high. TRPM6 was expressed at low levels in 5 out of 7 *β*-cell samples. TRPM5 was expressed at low level in only 2 of 7 *β*-cell samples. TRPM2 was expressed at low level in all the *β*-cell samples. TRPM1 and TRPM8 were not expressed in the *β*-cells.

Among the TRPV (transient receptor potential vanilloid) family, TRPV1, TRPV3, and TRPV6 were expressed in all samples of *β*-cells. TRPV2 was expressed at low levels in 4 out of 7 *β*-cell samples. Low-level expression of TRPV4 was observed in 2 out of 7 *β*-cell samples. TRPV5 was not expressed in the *β*-cells.

Among the TRPML (transient receptor potential mucolipin) family TRPML1 (MCOLN1), TRPML3 (MCOLN3) and TRPP1 (transient receptor potential polycistin 1, PKD2) were expressed at high levels in all the *β*-cell samples (Figure 1(b)). TRPP2 (transient receptor potential polycistin 2, PKD2L1) was expressed at low level in only 1 of 7 *β*-cell samples. TRPP3 (transient receptor potential polycistin 3, PKD2L2) was expressed at low level in 5 of 7 *β*-cell samples. The *β*-cells did not express TRPA1 (transient receptor potential ankyrin) and TRPML2 (MCOLN2) genes.

### 3.2. Expression of TRP Channel Genes in the *α*-Cells

TRPC1 and TRPC4 were expressed at high levels in all *α*-cell samples (Figure 1, top panel). TRPC5 and TRPC7 were expressed at low levels in all *α*-cells samples. TRPC6 was expressed at low level in 3 of 6 *α*-cell samples.

TRPM4 and TRPM7 were expressed at high levels in all *α*-cell samples. TRPM2 and TRPM6 were expressed at low levels in 1-2 *α*-cell samples. TRPM1, TRPM5, and TRPM8 were not expressed in these cells.

TRPV1, TRPV2, and TRPV3 were expressed in all *α*-cell samples. TRPV4 was expressed at low level in 3 of 6 *α*-cell samples. TRPV6 was also expressed at low levels in 4 of 6 *α*-cell samples. TRPV5 was not expressed in the *α*-cells.

TRPML1 (MCOLN1), TRPML3 (MCOLN3), and TRPP1 (PKD2) were expressed at high levels in all *α*-cell samples (Figure 1(a)). TRPA1 and TRPML2 (MNOLN2) were expressed at low level in only 1 of 6 *α*-cell samples. TRPP2 (PKD2L1) was not expressed in these cells. TRPP3 (PKD2L2) was expressed at low level in 4 of 6 *α*-cell samples.

### 3.3. Expression of the Isoforms of Selected TRP Channel Genes

**Figure 2:**
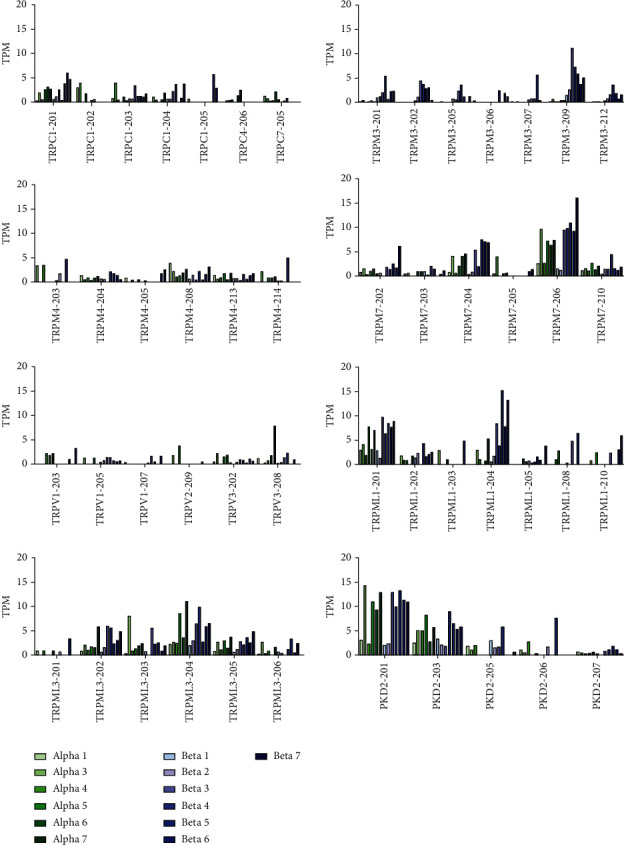
Expression of the isoforms of selected TRP channel genes in the purified *α*-cells and *β*-cells. Transcripts per million (TPM) values are shown as bar plots on linear scale. The figure shows the transcript levels of the isoforms of TRPC, TRPM3, TRPM4, TRPM7, TRPV, TRPML1, TRPML3, and PKD2. PKD2 = TRPP1. The *α*-cell preparations are shown in different shades of green bars and the *β*-cell preparations are shown in different shades of blue bars.

We analyzed the expression of the isoforms of 12 TRP channel genes: TRPC1, TRPC4, TRPC7, TRPM3, TRPM4, TRPM7, TRPV1, TRPV2, TRPV3, TRPML1 (MCOLN1), TRPML3 (MCOLN3), and PKD2 (TRPP1). Of the total 115 isoforms described for these selected genes, 50 were expressed in at least one of the cell populations.  Figure 2 shows the TPM values of the expressed isoforms in six *α*-cell- and seven *β*-cell preparations.

Four isoforms of the TRPC1 gene (TRPC1-201, TRPC1-202, TRPC1-203, and TRPC1-204) were expressed in these cells. The most abundant isoform of TRPC1 was TRPC1-201 (Figure 2(a)). The TRPC1-202 isoform was expressed mainly in the *α*-cells, whereas it was mostly absent in the *β*-cells. TRPC1-204 does not encode any protein. TRPC1-202 encodes a short protein, which probably does not form a channel. The only isoforms of TRPC4 and TRPC7 that were expressed were TRPC4-206 and TRPC7-205, respectively. Both were expressed mainly in the *α*-cells and were mostly absent in the *β*-cells. TRPC7-205 does not encode a protein.

Seven isoforms of TRPM3 (TRPM3-201, TRPM3-202, TRPM3-205, TRPM3-206, TRPM3-207, TRPM3-209, and TRPM3-212) were expressed in the *β*-cells. Of these, TRPM3-201 does not encode a protein. TRPM3-205, TRPM3-206, TRPM3-207, and TRPM3-212 encode short proteins that do not form channels. TRPM3 isoforms were mostly absent in the *α*-cells.

The isoforms of the other TRP channel genes were expressed in both the cell types. These are: six isoforms of TRPM4 (TRPM4-203, TRPM4-204, TRPM4-205, TRPM4-208, TRPM4-213, and TRPM4-214), six isoforms of TRPM7 (TRPM7-202, TRPM7-203, TRPM7-204, TRPM7-205, TRPM7-206, and TRPM7-210), three isoforms of TRPV1 (TRPV1-203, TRPV1-205, and TRPV1-207), one isoform of TRPV2 (TRPV2-209), two isoforms of TRPV3 (TRPV3-202, TRPV3-208), seven isoforms of TRPML1 or MCOLN1 (TRPML1-201, TRPML1-202, TRPML1-203, TRPML1-204, TRPML1-205, TRPML1-208, and TRPML1-210), six isoforms of TRPML3 or MCOLN3 (TRPML3-201, TRPML3-202, TRPML3-203, TRPML3-204, TRPML3-205, and TRPML3-206), and five isoforms of PKD2 or TRPP1 (PKD2-201, PKD2-203, PKD-205, PKD2-206, and PKD2-207). Of these, the following isoforms do not encode any protein: TRPM4-204, TRPM4-214, TRPM7-202, TRPM7-203, TRPV1-205, TRPV2-209, TRPV3-208, TRPML1-202, TRPML1-203, TRPML1-204, TRPML1-205, TRPML1-210, TRPML3-204, TRPML3-205, TRPP1-203, TRPP1-206, and TRPP1-207.  Table 1 shows a summary of the TRP channel genes and their isoforms that were expressed in each of the cell preparations.

### 3.4. Differential Expression of TRP Channel Genes and their Isoforms in the *α*- and *β*-Cells

**Figure 3:**
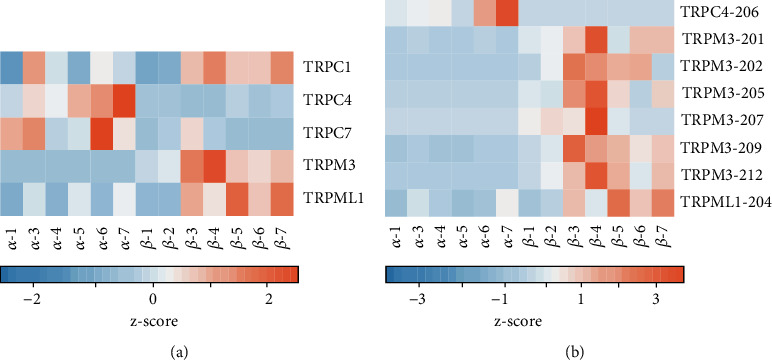
Differentially expressed TRP channel genes and their isoforms in the purified human *α*-cells and *β*-cells. (a) Differentially expressed TRP channel genes, TRPC1, TRPM3 and TRPML1 showed higher expression in the *β*-cells, while TRPC4 and TRPC7 showed higher expression in the *α*-cells. (b) Differentially expressed isoforms of TRP channel genes, TRPC4-206 showed higher expression in *α*-cells, while TRPM3-201, TRPM3-202, TRPM3-205, TRPM3-207, TRPM3-209, TRPM3-212, and TRPML1-204 showed higher expression in the *β*-cells. The color indicates the relative expression of each gene per row. Lower expression is represented in shades of blue, while higher expression is represented in shades of orange.

We analyzed the RNA-seq data to identify the TRP channel genes and the isoforms that were expressed differentially in the *α*-cells and *β*-cells of the human islets of Langerhans. We found that of the 27 TRP genes, five (*TRPC1*, *TRPC4*, *TRPC7*, *TRPM3*, and *TRPML1*) were differentially expressed in these two cell types. Compared to the *α*-cells, the *β*-cells expressed *TRPC1* (1.5-fold, FDR < 0.01), *TRPM3* (25-fold, FDR < 0.01), and *TRPML1* (1.8-fold, FDR < 0.01) at a higher level. *TRPC4* (22.8-fold, FDR < 0.01) and *TRPC7* (7.0-fold, FDR < 0.01) were expressed at a higher level in the *α*-cells than in the *β*-cells (Figure 3(a)).

Of the 215 isoforms described in the human genome for the TRP channel genes, six *TRPM3*, one *TRPML1*, and one *TRPC4* isoform were differentially expressed in these two cell types (Figure 3(b)). In the *β*-cells, the expression of the following transcripts of *TRPM3* and *TRPML1* were higher compared to those in the *α*-cells: *TRPM3-201* (23.3-fold higher, FDR < 0.01), *TRPM3-202* (200-fold higher, FDR < 0.01), *TRPM3-205* (20-fold higher, FDR < 0.01), *TRPM3-207* (14.5-fold higher, FDR < 0.01), *TRPM3-209* (20.8-fold higher, FDR < 0.01), *TRPM3-212* (16.6-fold higher, FDR < 0.01), and *TRPML1-204* (3.7-fold higher, FDR < 0.01). The *TRPC4-206* isoform was expressed at a 45-fold higher (FDR < 0.01) level in the *α*-cells compared to the *β*-cells (Figure 3(b)).

## 4. Discussion

By analyzing RNA-seq data obtained from highly purified *α*-cells and *β*-cells of human islets of Langerhans, we have identified the TRP channel genes and their isoforms that are differentially expressed in these two cell types. The differentially expressed genes were as follows: *TRPC1, TRPC4, TRPC7, TRPM3*, and *TRPML1*. *TRPM3*, *TRPC1*, and *TRPML1* were expressed at a higher level in the *β*-cells, whereas *TRPC4* and *TRPC7* were expressed at a higher level in the *α*-cells. *TRPM3-201*, *TRPM3-202*, *TRPM3-205*, *TRPM3-207*, *TRPM3-209*, *TRPM3-212*, and *TRPML1-204* isoforms were expressed at a higher level in the *β*-cells, whereas the *TRPC4-206* isoform was expressed at a higher level in the *α*-cells (Figure 1(b)). Higher level of expression of *TRPC1* and *TRPM3* in the *β*-cells is consistent with the roles of these channels in the regulation of insulin secretion [17, 18].

### 4.1. TRP Channel Genes in the *α*-Cells

We have identified for the first time the TRP channel genes that are expressed in the human *α*-cells. We found that the *α*-cells expressed *TRPC1*, *TRPC4*, *TRPM4*, *TRPM7*, *TRPV1*, *TRPV2*, *TRPV3*, *TRPML1*, *TRPML3*, and *TRPP1* at relatively high level. These cells expressed *TRPC5*, *TRPC7*, *TRPC6*, *TRPM2*, *TRPM6*, *TRPV4*, *TRPV6*, *TRPA1*, *TRPML2*, and *TRPP3* at relatively low levels. The *α*-cells did not express *TRPC3*, *TRPM1*, *TRPM5*, *TRPM8*, *TRPV5*, and *TRPP2*.

### 4.2. TRP Channel Genes in the *β*-Cells

The *β*-cells expressed *TRPC1*, *TRPM3*, *TRPM4*, *TRPM7*, *TRPV1*, *TRPV3*, *TRPV6*, *TRPML1*, *TRPML3*, and *TRPP1* at relatively high level. These cells expressed *TRPC4*, *TRPC5*, *TRPC6*, *TRPC7*, *TRPM2*, *TRPM5*, *TRPM6*, *TRPV2*, *TRPV4*, *TRPP2*, and *TRPP3* at relatively low level. The *β*-cells did not express *TRPC3*, *TRPM1*, *TRPM8*, *TRPV5*, *TRPA1*, and *TRPML2*.

The expression profile of the TRP channel genes in the *β*-cells reported in the present study is different from that reported in an earlier report [12]. It was reported that the *β*-cells do not express *TRPM5*, *TRPM6*, *TRPV1*, *TRPV2*, *TRPV3*, *TRPV4*, and *TRPV6* [12]. We found that these genes are expressed, albeit at low level, in at least some *β*-cell preparations. In the previous study, we analyzed only two *β*-cell samples; in the present study, we have analyzed seven *β*-cell samples. In the previous study, we used an arbitrary cutoff value of fragments per kilobase million (FPKM) > 1 as an expression threshold to decide whether a gene is expressed or not. Because of using such a cutoff, genes that were expressed at relatively low levels were stated as “not expressed.” In the present study, we have reported the results as transcripts per kilobase Million (TPM) and we did not use any arbitrary cutoff value. Thus, we have identified all the TRP channel genes including the ones that are expressed at low levels in these cells.

### 4.3. Functional Implications of TRP Channel Gene Expression in the *β*-Cells

Expression of *TRPC1*, *TRPM3*, *TRPM4*, *TRPM2*, and *TRPM7* in the *β*-cells is consistent with the reports that the TRPC1, TRPM3, TRPM4, TRPM2, and TRPM7 channels are involved in regulating insulin secretion [18–24]. In *β*-cells, TRPC1 together with Orai1 forms the pore-forming subunit of the store-operated Ca^2+^ channel, which plays important role in mediating glucose-stimulated insulin secretion [17]. TRPM7 channel, which is permeable to Ca^2+^, Mg^2+^ and Zn^2+^ is highly expressed in human *β*-cells [12]. It has been reported that knockdown of *TRPM7* increases insulin secretion presumably by decreasing the cytoplasmic Mg^2+^ concentration [24]. TRPM7 is also involved in mediating *β*-cell proliferation [25].

TRPM4 and TRPM5 are closely related nonselective cation channels activated by [Ca^2+^]*_i_*. From studies in rodents, it is known that both ion channels are involved in mediating Ca^2+^ signaling and insulin secretion [22, 26–28]. Consistent with our previous study, we have found that human *β*-cells express essentially only the *TRPM4* suggesting that the TRPM4 channels are more important than the TRPM5 channels in mediating insulin secretion from human *β*-cells [12]. There are numerous examples of similar differences between the mouse and the human *β*-cells [11].

Among the thermosensitive TRP channels TRPM3 is most abundant in the *β*-cells. Expression of *TRPM3* in the *β*-cells was 25-fold higher compared to that in the *α*-cells. One isoform of *TRPM3* (*TRPM3-202)* was highly expressed in the *β*-cells (200-fold higher compared to the *α*-cells). This isoform is highly homologous to the mouse mTRPM3a4 [29]. Another isoform of TRPM3, namely, the *TRPM3-209* was expressed at a level 20.8 times higher in the *β*-cells than in the *α*-cells. This isoform is homologous to the mouse mTRP3b2 [30]. Previous studies have shown that TRPM3, which is activated by pregnenolone, contribute to insulin secretion from the *β*-cells by multiple mechanisms [31, 32]. The other four thermosensitive TRP channel genes expressed in the *β*-cells were *TRPV1*, *TRPV3*, *TRPV2*, and *TRPM2*, but their expression was much lower (<4 TPM) compared to the expression of *TRPM3*.

Consistent with a previous report, we could not detect expression of *TRPA1*, *TRPV5*, *TRPM1*, and *TRPM8* in the human *β*-cells [12]. It has been reported that TRPA1 channel is involved in insulin secretion from rat and human *β*-cells [33, 34], but our results do not support this view since we found that the human *β*-cells do not express *TRPA1*. According to one report TRPV5 (also called ECaC1) channel is abundantly expressed in rat *β*-cells [35], but our results show that TRPV5 is not expressed in human *β*-cells.

### 4.4. Functional Implications of TRP Channel Gene Expression in the *α*-Cells

Only a few studies have investigated the roles of TRP channels in *α*-cell function. Human *α*-cells express TRPM4 but not the TRPM5. Activation of TRPM4 channel depolarizes membrane potential, increases cytoplasmic free Ca^2+^ concentration and stimulates glucagon secretion [36]. Glucagon secretagogues like arginine vasopressin (AVP) activate a G_q_-coupled receptor leading to the production of inositol 1,4,5-trisphosphate and release of Ca^2+^ from the endoplasmic reticulum (ER). Emptying of the ER triggers Ca^2+^-entry through the store-operated Ca^2+^ channels. Ca^2+^ activates the TRPM4, and Na^+^-influx through the TRPM4 channels and depolarizes the membrane potential. This leads to the activation of the voltage gated Ca^2+^ channels and further elevation of the cytoplasmic free Ca^2+^ concentration, which triggers glucagon release [36]. It should be noted that TRPM4 is expressed in both human *α*-cells and *β*-cells and it may be involved in mediating the secretion of both glucagon and insulin [37].

*TRPM7* is also expressed at high level in the *α*-cells. TRPM7 channels are involved in early pancreatic endocrine development as evidenced from the fact that ablation of *TRPM7* in mice reduces total *α*-cell mass [25].

### 4.5. Some General Comments

This study is based on *β*-cells and *α*-cells obtained from nondiabetic donors aged 4-60 years. For understanding the roles of TRP channels in adaptations and dysfunctions of these cells, it will be necessary to obtain such cells from enough donors of different age groups and from donors who have conditions like impaired glucose tolerance or diabetes. Our results show heterogeneity of expression of TRP channel genes, and we speculate that such heterogeneity could be related to the heterogeneity of the endocrine cells of the islets [38]. We have reported the expression of the TRP channel genes only, but it remains unclear whether these genes lead to the expression of proteins and if those proteins form functional ion channels. More functional studies will be needed to validate the present findings and elaborate the remaining issues further.

## 5. Conclusions

We have identified the TRP channel genes that are expressed in the human *α*-cells and the *β*-cells. We have found that *TRPC1*, *TRPC4*, *TRPC7*, *TRPM3*, and *TRPML1* are differentially expressed in these two cell types. *TRPC4* and *TRPC7* are more expressed in the *α*-cells than in the *β*-cells, but the functions of these two channels in the *α*-cells have not been reported. *TRPM3-202* was expressed at 200-fold higher level in the *β*-cells but the regulation of this channel has not been reported. Our findings about the expression patterns of the different TRP channel genes and their isoforms may contribute to the understanding of the physiological roles of these channels in the regulation of hormone secretion from these cells and other functions of these cells.

## Figures and Tables

**Table 1:** Summary of expression of TRP genes and their isoforms.

Gene	Isoforms	Differentially expressed (FDR < 0.01)	Expression in *α*-cells	Expression in *β*-cells
TRPA1	TRPA1-201, TRPA1-202, TRPA1-203, TRPA1-204, TRPA1-205, TRPA1-206	*ns*	nd	nd
TRPC1	TRPC1-201, TRPC1-202, TRPC1-203, TRPC1-204	In *β*-cells Gene: 1.5 FC Isoforms: *ns*	TRPC1-201, TRPC1-202^∗^, TRPC1-203, TRPC1-204	TRPC1-201, TRPC1-202, TRPC1-203, TRPC1-204
TRPC3	TRPC3-201, TRPC3-202, TRPC3-203, TRPC3-204, TRPC3-205	*ns*	nd	nd
TRPC4	TRPC4-201, TRPC4-202, TRPC4-203, TRPC4-204, TRPC4-205, TRPC4-206, TRPC4-207, TRPC4-208, TRPC4-209, TRPC4-210	In *α*-cells: Gene: 22.8 FC Isoforms: TRPC4-206 45.0 FC	TRPC4-206	TRPC4-206∗
TRPC5	TRPC5-201	*ns*	Low	Low
TRPC6	TRPC6-201, TRPC6-202, TRPC6-203, TRPC6-204, TRPC6-205, TRPC6-206, TRPC6-207	*ns*	Low	Low
TRPC7	TRPC7-201, TRPC7-202, TRPC7-203, TRPC7-204, TRPC7-205, TRPC7-206, TRPC7-207	In *α*-cells Gene: 7.0 FC Isoforms: *ns*	TRPC7-205	TRPC7-205∗
TRPM1	TRPM1-201, TRPM1-202, TRPM1-203, TRPM1-204, TRPM1-205, TRPM1-206, TRPM1-207, TRPM1-208, TRPM1-209, TRPM1-210, TRPM1-211, TRPM1-212	*ns*	nd	nd
TRPM2	TRPM2-201, TRPM2-202, TRPM2-203, TRPM2-204, TRPM2-205, TRPM2-206, TRPM2-207, TRPM2-208	*ns*	Low	Low
TRPM3	TRPM3-201, TRPM3-202, TRPM3-203, TRPM3-204, TRPM3-205, TRPM3-206, TRPM3-207, TRPM3-208, TRPM3-209, TRPM3-210, TRPM3-211, TRPM3-212, TRPM3-213, TRPM3-214, TRPM3-215, TRPM3-216	In *β*-cells Gene: 25.0 FC Isoforms: TRPM3-201 23.3 FC; TRPM3-202 200.0 FC; TRPM3-205 20.0 FC; TRPM3-207 14.5 FC; TRPM3-209 20.8 FC; TRPM3-212 16.6 FC	TRPM3-201^∗^, TRPM3-202^∗^, TRPM3-205^∗^, TRPM3-206^∗^, TRPM3-207^∗^, TRPM3-209^∗^, TRPM3-212^∗^	TRPM3-201, TRPM3-202, TRPM3-205, TRPM3-206, TRPM3-207, TRPM3-209, TRPM3-212
TRPM4	TRPM4-201, TRPM4-202, TRPM4-203, TRPM4-204, TRPM4-205, TRPM4-206, TRPM4-207, TRPM4-208, TRPM4-209, TRPM4-210, TRPM4-211, TRPM4-212, TRPM4-213, TRPM4-214	*ns*	TRPM4-203, TRPM4-204, TRPM4-205, TRPM4-208, TRPM4-213, TRPM4-214	TRPM4-203, TRPM4-204, TRPM4-205, TRPM4-208, TRPM4-213, TRPM4-214
TRPM5	TRPM5-201, TRPM5-202, TRPM5-203, TRPM5-204	*ns*	Low	Low
TRPM6	TRPM6-201, TRPM6-202, TRPM6-203, TRPM6-204, TRPM6-205	*ns*	Low	Low
TRPM7	TRPM7-201, TRPM7-202, TRPM7-203, TRPM7-204, TRPM7-205, TRPM7-206, TRPM7-207, TRPM7-208, TRPM7-209, TRPM7-210	*ns*	TRPM7-202, TRPM7-203, TRPM7-204, TRPM7-205, TRPM7-206, TRPM7-210	TRPM7-202, TRPM7-203, TRPM7-204, TRPM7-205, TRPM7-206, TRPM7-210
TRPM8	TRPM8-201, TRPM8-202, TRPM8-203, TRPM8-204, TRPM8-205, TRPM8-206, TRPM8-207, TRPM8-208, TRPM8-209, TRPM8-210, TRPM8-211, TRPM8-212, TRPM8-213, TRPM8-214	*ns*	nd	nd
TRPV1	TRPV1-201, TRPV1-202, TRPV1-203, TRPV1-204, TRPV1-205, TRPV1-206, TRPV1-207, TRPV1-208, TRPV1-209, TRPV1-210	*ns*	TRPV1-203, TRPV1-205, TRPV1-207	TRPV1-203, TRPV1-205, TRPV1-207
TRPV2	TRPV2-201, TRPV2-202, TRPV2-203, TRPV2-204, TRPV2-205, TRPV2-206, TRPV2-207, TRPV2-208, TRPV2-209	*ns*	TRPV2-209	TRPV2-209
TRPV3	TRPV3-201, TRPV3-202 TRPV3-203, TRPV3-204, TRPV3-205, TRPV3-206, TRPV3-207, TRPV3-208, TRPV3-209, TRPV3-210, TRPV3-211	*ns*	TRPV3-202, TRPV3-208	TRPV3-202, TRPV3-208
TRPV4	TRPV4-201, TRPV4-202, TRPV4-203, TRPV4-204, TRPV4-205, TRPV4-206, TRPV4-207, TRPV4-208	*ns*	Low	Low
TRPV5	TRPV5-201, TRPV5-202, TRPV5-203	*ns*	nd	nd
TRPV6	TRPV6-201, TRPV6-202, TRPV6-203, TRPV6-204, TRPV6-205, TRPV6-206, TRPV6-207, TRPV6-208, TRPV6-209	*ns*	Low	Low
TRPML1	TRPML1-201, TRPML1-202, TRPML1-203, TRPML1-204, TRPML1-205, TRPML1-206, TRPML1-207, TRPML1-208, TRPML1-209, TRPML1-210	In *β*-cells Gene: 1.8 FC Isoforms: TRPML1-204 3.7 FC	TRPML1-201, TRPML1-202, TRPML1-203, TRPML1-204, TRPML1-205, TRPML1-208, TRPML1-210	TRPML1-201, TRPML1-202, TRPML1-203, TRPML1-204, TRPML1-205, TRPML1-208, TRPML1-210
TRPML2	TRPML2-201, TRPML2-202, TRPML2-203, TRPML2-204, TRPML2-205, TRPML2-206	*ns*	nd	nd
TRPML3	TRPML3-201, TRPML3-202, TRPML3-203, TRPML3-204, TRPML3-205, TRPML3-206	*ns*	TRPML3-201, TRPML3-202, TRPML3-203, TRPML3-204, TRPML3-205, TRPML3-206	TRPML3-201, TRPML3-202, TRPML3-203, TRPML3-204, TRPML3-205, TRPML3-206
PKD2	PKD2-201, PKD2-202, PKD2-203, PKD2-204, PKD2-205, PKD2-206, PKD2-207	*ns*	PKD2-201, PKD2-203, PKD-205, PKD2-206, PKD2-207	PKD2-201, PKD2-203, PKD-205, PKD2-206, PKD2-207
PKD2L1	PKD2L1-201, PKD2L1-202, PKD2L1-203, PKD2L1-204	*ns*	nd	nd
PKD2L2	PKD2L2-201, PKD2L2-202, PKD2L2-203, PKD2L2-204, PKD2L2-205, PKD2L2-206, PKD2L2-207	*ns*	Low	Low

Legend: ns: not significant; FC: fold-change; nd: not detected (mean TPM < 0.5); ^∗^mostly absent in this cell population.

## Data Availability

The study used RNA sequence data published by Blodget et al., and this has been cited as reference [6] in the text.
